# Drug-cost trajectories after national volume-based procurement in Chinese public hospitals: a 31-province interrupted time-series analysis

**DOI:** 10.3389/fmed.2026.1970115

**Published:** 2026-09-30

**Authors:** Jing Mao, Guangqi Liu

**Affiliations:** Shanghai Sixth People’s Hospital Affiliated to Shanghai Jiao Tong University School of Medicine, Shanghai, China

**Keywords:** China, drug costs, interrupted time series, public hospitals, revenue structure, volume-based procurement

## Abstract

**Background:**

China's national volume-based procurement (NVBP) policy reduced prices for selected medicines, but national aggregate drug-cost trends are exposed to concurrent reforms and nonlinear prepolicy trajectories. We assessed public-hospital drug-cost trajectories around 2019 and explored hospital revenue structure.

**Methods:**

This uncontrolled ecological interrupted time-series study used a balanced panel of 31 province-level units from 2011 through 2024 (434 province-years). Province fixed-effects segmented regressions estimated immediate level and annual trend changes in per-encounter drug costs and drug-cost shares. Sensitivity analyses examined nonlinear and piecewise pretrends, non-drug costs as a control series, calendar-year fixed effects in drug-versus-non-drug comparative models, consumer-price-index deflation, multiple testing, and influential years. Revenue structure was described in 35 observations from 4 data-available provinces, with wild cluster bootstrap inference reported only as exploratory.

**Results:**

No primary outcome showed a clear immediate 2019 level change (all *P* > .05). Under the prespecified single-linear-pretrend model, annual trends changed by −1.67 Chinese yuan (CNY) per outpatient visit [95% confidence interval (CI), −2.70 to −0.63] and −164.53 CNY per inpatient discharge (95% CI, −199.80 to −129.26). In the linear model, the model-implied 2024 differences were −5.31 CNY per outpatient visit (95% CI, −11.70 to 1.07; *P* = .100) and −753.18 CNY per inpatient discharge (95% CI, −924.76 to −581.60; *P* < .001). However, results were highly sensitive to pretrend form: with a 2016-2018 piecewise pretrend, corresponding changes were 1.64 CNY (95% CI, 0.27 to 3.02) and 17.11 CNY (95% CI, −33.46 to 67.68). Inpatient non-drug cost also changed substantially (−409.50 CNY/year; 95% CI, −483.03 to −335.97), and the inpatient drug-versus-non-drug differential trend change was not significant after calendar-year fixed effects (*P* = .488). Drug-cost shares remained below 2011 levels but declined more slowly after 2019 in all specifications. The 4-province revenue trend-change interval crossed zero with wild cluster bootstrap inference (*P* = .055). Conclusion: The single-linear ITS suggested post-2019 changes in aggregate drug-cost trajectories, but nonlinear pretrends and synchronous non-drug changes materially weakened their specificity to NVBP. These annual ecological data support temporal description, not attribution of the observed breakpoint to NVBP alone.

## 1 Introduction

Public hospitals deliver most specialist and inpatient care in China and remain central to the country's efforts to contain health expenditure while maintaining access and quality. Since the 2009 health-system reform, public-hospital financing has been reshaped through removal of drug markups, adjustments to medical-service prices, reforms to provider payment, and changes in government subsidies (1, 2). These reforms reduced the formal role of medicine sales in hospital financing, but they also created a continuing need to monitor patient drug costs and the financial structure of hospitals.

China introduced national volume-based procurement (NVBP) to pool purchasing power, link tendered prices to committed procurement volumes, promote quality-assured generics, and reduce medicine costs. The initial “4 + 7” pilot covered 11 cities and specified committed purchasing volumes equal to approximately 60% to 70% of prior use for selected medicines; implementation began in 2019 and was subsequently expanded nationwide (3, 4). Evidence based on procurement records has reported large reductions in winning-bid prices and expenditures, increased use of selected products, and possible substitution toward nonwinning or alternative medicines (5–9). However, the 25 molecules in the first pilot accounted for only about 2% of hospital-channel sales in the pilot cities, whereas the first 6 procurement batches together later involved drug purchasing amounts equivalent to approximately 30% of annual public-medical-institution drug procurement (10, 11). This change in coverage makes it implausible to assume that an aggregate 2019 breakpoint represents NVBP alone.

A recent panel interrupted time-series (ITS) study used national statistical yearbooks to examine broad health-cost and utilization indicators through 2022 (12). Separately, decomposition of national and provincial public-hospital expenditures showed that service volume was a major driver of expenditure growth, underscoring that per-encounter prices and total spending answer different policy questions (13). The 2019 breakpoint also coincided with national reforms outside NVBP: a 30-city diagnosis-related-group (DRG) payment pilot began in 2019, 71 diagnosis-intervention-packet (DIP) pilots were underway by 2020, negotiated insurance-list access expanded, and a new government accounting system took effect (14–17). Earlier zero-markup and medical-service-price reforms were still shaping pre-2019 trajectories (1, 2, 18–20). It therefore remains uncertain whether broad changes in public-hospital drug costs around 2019 were specific to NVBP or reflected wider system change.

We therefore evaluated 4 outcomes in public hospitals across 31 province-level administrative units from 2011 through 2024: outpatient drug cost per visit, inpatient drug cost per discharge, outpatient drug-cost share, and inpatient drug-cost share. We prespecified a single-linear-pretrend ITS for comparability with prior yearbook research but treated robustness to nonlinear pretrends and synchronous non-drug costs as central to interpretation. As a secondary objective, we described drug income as a share of medical income in 4 provinces selected solely because exact-scope longitudinal data were available. Because the intervention was national and no concurrent untreated group was available, all estimates were interpreted as temporal associations rather than isolated causal effects.

## 2 Methods

### 2.1 Study design and reporting

We conducted an uncontrolled ecological ITS study using annual province-level panel data. ITS uses the preintervention trajectory as the counterfactual and estimates whether an intervention coincides with a level change, a slope change, or both (21–23). The study was reported with reference to the STROBE statement and methodological recommendations for ITS evaluations (23, 24). The unit of analysis was the province-year.

### 2.2 Policy intervention and breakpoint

The General Office of the State Council issued the national centralized drug procurement and use pilot program in January 2019. The program selected 11 pilot cities, required public medical institutions to prioritize selected medicines, and linked negotiated prices to committed procurement volume (3). Implementation began in pilot cities during 2019, followed by expansion to all provinces. We prespecified 2019 as the main breakpoint to represent the start of national policy implementation and expansion. Because annual statistics cannot distinguish implementation month and nationwide uptake continued into 2020, we used 2020 as an alternative breakpoint in sensitivity analyses.

### 2.3 Data sources and study sample

The main analysis used publicly available annual tables from the China Health Statistical Yearbooks for calendar years 2011 through 2024 (25). The tables report province-level average outpatient and inpatient medical costs in public hospitals and their drug components. We included the 31 province-level administrative units in mainland China; Hong Kong, Macao, and Taiwan were outside the statistical scope. Data were extracted into a standardized panel, independently checked against the source tables, and subjected to range, duplication, and accounting-identity checks. The resulting main panel was balanced, with 31 provinces observed in each of 14 years (434 province-year observations). No missing outcome was imputed, and no simulated value was used in any empirical analysis.

The exploratory revenue-structure dataset was assembled from official provincial health statistical yearbooks and related official statistical publications for Chongqing, Guangdong, Gansu, and Sichuan. It contained 41 source-backed observations from 2012 through 2024. The main exploratory analysis retained 35 observations whose statistical scope corresponded exactly to public hospitals: Chongqing, 2016–2024; Guangdong, 2018–2024; Gansu, 2016–2022; and Sichuan, 2012–2023. Six Guangdong observations from 2012 through 2017 described a narrower legacy scope of government-run nonprofit hospitals and were used only in a scope-sensitivity analysis.

### 2.4 Outcomes

The 4 primary drug-cost burden outcomes were:

1. Outpatient drug cost per visit, in nominal CNY.
2. Inpatient drug cost per discharge, in nominal CNY.
3. Outpatient drug cost divided by total outpatient medical cost per visit, expressed as a percentage.
4. Inpatient drug cost divided by total inpatient medical cost per discharge, expressed as a percentage.

Primary cost outcomes were analyzed in nominal CNY because these are the amounts recorded in the yearbooks. As a sensitivity analysis, we converted them to constant 2019 CNY using China's annual consumer price index (CPI; 2010 = 100) from the World Bank, which reports International Monetary Fund International Financial Statistics as its source (26). Cost shares measure the drug component within the encounter-level medical bill and are reported as percentage points. These outcomes do not measure total provincial drug expenditure, medicine volume, insurance reimbursement, or patient out-of-pocket spending.

The exploratory revenue-structure outcome was drug income divided by medical income and expressed as a percentage. This ratio measures public hospitals' recorded dependence on drug income within medical income, not profit from medicines. Secondary revenue and expenditure ratios were described only in the supplement because their availability differed across provinces and years.

### 2.5 Main statistical analysis

We summarized each main outcome by calendar year using the unweighted mean and standard deviation across 31 provinces. The primary segmented model was:

Y_it = alpha_i + beta_1(time_t) + beta_2(post2019_t) + beta_3(time_after2019_t) + error_it

where alpha_i denotes province fixed effects; time_t was coded 0 in 2011 and increased by 1 each year; post2019_t equaled 1 from 2019 onward; and time_after2019_t equaled 0 through 2019 and increased by 1 in each subsequent year. Thus, beta_1 estimated the annual pre-2019 trend, beta_2 the immediate level change in 2019, and beta_3 the change in annual trend after 2019. The post-2019 annual trend was beta_1 + beta_3. The model-implied difference in 2024 from continuation of the pre-2019 trend was beta_2 + 5(beta_3).

Standard errors were clustered by province to allow heteroskedasticity and within-province dependence. Complete calendar-year fixed effects cannot be included in the absolute ITS because a nationwide intervention indicator and slope change that vary only by year are collinear with year indicators. Consequently, the primary model cannot control for other national shocks at the 2019 breakpoint. To examine specificity, we fitted separate outpatient and inpatient comparative models that stacked drug and non-drug costs, included province-by-component and full calendar-year fixed effects, and estimated the differential drug-versus-non-drug level and trend changes with 2-way province-and-year clustered standard errors. These models absorb common annual shocks but estimate relative component changes rather than the absolute NVBP association. We report coefficients with 95% CIs and 2-sided *P* values. Benjamini-Hochberg false-discovery-rate (FDR) corrections were calculated separately across the 4 primary outcomes for the immediate-change and trend-change families.

### 2.6 Sensitivity, diagnostic, and heterogeneity analyses

We evaluated an alternative 2020 breakpoint; added an indicator for the 2020–2022 coronavirus disease 2019 (COVID-19) period; excluded 2020–2022; included province-specific linear trends; log-transformed the 2 cost outcomes; and sequentially omitted each province. In direct response to pretrend concerns, we additionally fitted a quadratic baseline-time specification and a piecewise specification allowing the 2016–2018 slope to differ from the earlier pretrend. We constructed per-visit and per-discharge non-drug costs as total medical cost minus drug cost and analyzed them as control series. CPI-deflated costs, leave-one-year-out analyses, and the comparative calendar-year-fixed-effects models provided further checks. Because diagnostics indicated residual serial correlation and cross-sectional dependence, we also calculated Driscoll-Kraay, 2-way clustered, and annual-mean Newey-West standard errors (27). Specific diagnostic statistics and *P* values are reported in the supplement.

Regional heterogeneity was explored for Eastern, Central, Western, and Northeastern China by interacting region with the pre-2019 trend, immediate change, and trend change. Joint Wald tests assessed whether the post-2019 trend-change coefficients differed by region. Regional estimates were exploratory, especially for Northeastern China, which contained 3 provinces.

For the revenue-structure outcome, the main text presents only observed province trajectories. The full province fixed-effects segmented model is reported in the supplement using conventional province-clustered inference and wild cluster bootstrap-t confidence intervals with Webb weights, null imposition, and 9,999 replications. The 4 provinces were selected solely because exact-scope longitudinal public-hospital revenue data were available. With 35 observations and only 4 clusters, unequal observation periods, and an accounting-system change exactly at the 2019 breakpoint, this analysis was defined as descriptive and exploratory rather than confirmatory. Missing revenue outcomes were not imputed.

All analyses were performed in R version 4.5.3 (R Foundation for Statistical Computing, Vienna, Austria), principally using fixest version 0.14.2, sandwich version 3.1–2, and nlme version 3.1–168.

### 2.7 Ethics

The study used only publicly available, aggregate province-level statistics and contained no individual-level or identifiable information. Institutional ethics review and informed consent were therefore not applicable.

## 3 Results

### 3.1 Main panel and descriptive trajectories

The main dataset contained all 31 provinces for every year from 2011 through 2024, yielding 434 province-year observations with no missing values for the primary or constructed non-drug outcomes. Across provinces, mean outpatient drug cost per visit increased from 85.60 CNY [standard deviation (SD), 34.70] in 2011 to 126.71 CNY (SD, 42.71) in 2024. Mean inpatient drug cost per discharge decreased from 2951.52 CNY (SD, 980.09) to 2047.97 CNY (SD, 820.42). Mean outpatient drug-cost share declined from 49.42% (SD, 5.74%) to 37.27% (SD, 4.06%), and mean inpatient drug-cost share declined from 42.46% (SD, 3.87%) to 19.73% (SD, 2.34%) (Table 1 and Figure 1). Table 1 reports every annual mean rather than selected years.

**Table 1:** Annual provincial mean public-hospital drug costs and cost shares, China, 2011-2024.

Year	Outpatient drug cost per visit, mean (SD), CNY	Inpatient drug cost per discharge, mean (SD), CNY	Outpatient drug-cost share, mean (SD), %	Inpatient drug-cost share, mean (SD), %
2011	85.60 (34.70)	2951.52 (980.09)	49.42 (5.74)	42.46 (3.87)
2012	92.06 (35.54)	3,097.30 (1,013.59)	49.38 (5.53)	41.71 (3.83)
2013	98.21 (35.26)	3,225.69 (1,003.75)	48.30 (5.26)	40.15 (3.73)
2014	103.86 (35.50)	3,283.94 (1,002.03)	48.00 (5.30)	39.05 (3.52)
2015	108.20 (37.12)	3,366.06 (1,071.80)	46.73 (5.24)	37.71 (3.77)
2016	109.60 (37.19)	3,310.50 (1,035.66)	45.44 (5.04)	35.26 (3.55)
2017	108.01 (35.98)	3,007.21 (891.35)	42.87 (4.28)	31.14 (3.51)
2018	109.97 (35.04)	2,823.81 (852.50)	41.34 (4.16)	27.91 (2.80)
2019	116.97 (34.33)	2,904.83 (885.69)	41.42 (4.11)	27.30 (2.80)
2020	127.01 (47.61)	3,045.55 (1,062.39)	39.79 (4.51)	26.06 (2.83)
2021	120.87 (41.88)	2,951.24 (1,016.95)	38.20 (4.28)	24.84 (2.97)
2022	128.77 (44.23)	2,845.28 (1,008.76)	38.93 (4.39)	24.24 (3.14)
2023	128.80 (42.70)	2,449.49 (916.91)	37.52 (4.16)	22.36 (2.77)
2024	126.71 (42.71)	2,047.97 (820.42)	37.27 (4.06)	19.73 (2.34)

Each year contains 31 province-level administrative units. Values are unweighted means across provinces.

Costs are nominal currency amounts. CNY, Chinese yuan; SD, standard deviation.

**Figure 1:**
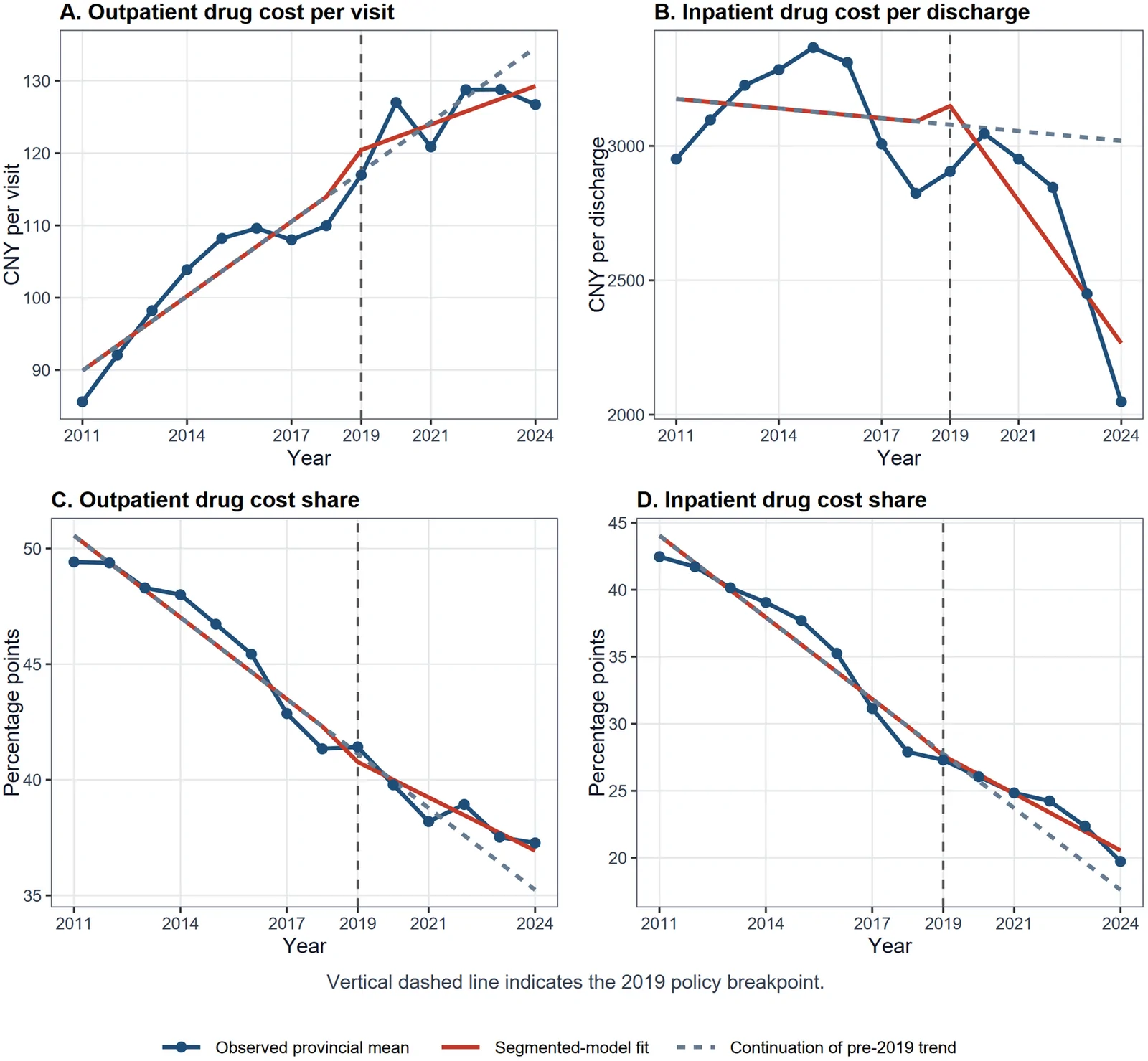
Observed and model-estimated public-hospital drug-cost burden in 31 Chinese provinces, 2011–2024. Panels show (A) outpatient drug cost per visit, (B) inpatient drug cost per discharge, (C) outpatient drug-cost share, and (D) inpatient drug-cost share. Points and dark-blue lines show unweighted annual province means. Red lines show fitted values from province fixed-effects segmented regressions. Blue-gray dashed lines show continuation of the estimated pre-2019 trend. The vertical dashed line marks the 2019 policy breakpoint. Costs are nominal CNY; shares are percentage points.

The observed series showed that both drug-cost shares were already declining before 2019. Inpatient drug cost per discharge also declined during much of the prepolicy period, whereas outpatient drug cost per visit generally increased. The segmented models therefore evaluated changes relative to these existing trajectories rather than simple pre-post differences.

### 3.2 Segmented regression of drug-cost burden

There was no clear immediate 2019 level change in any primary outcome (Table 2 and Figure 2). The immediate change was 3.02 CNY (95% CI, −0.29 to 6.32; *P* = .072) for outpatient drug cost per visit and 69.46 CNY (95% CI, −3.84 to 142.75; *P* = .062) for inpatient drug cost per discharge. Immediate changes in outpatient and inpatient drug-cost shares were −0.36 (95% CI, −0.99 to 0.26; *P* = .246) and −0.15 (95% CI, −0.78 to 0.47; *P* = .626) percentage points, respectively.

**Table 2:** Segmented regression estimates for public-hospital drug-cost burden at the 2019 policy breakpoint.

Outcome	Pre-2019 annual trend	Immediate 2019 change	Annual trend change	Post-2019 annual trend	Difference in 2024
Outpatient drug cost per visit	3.44 (2.88 to 3.99); *P* < .001	3.02 (−0.29 to 6.32); *P* = .072	−1.67 (−2.70 to −0.63); *P* = .003	1.77 (0.93 to 2.62); *P* < .001	−5.31 (−11.70 to 1.07); *P* = .100
Inpatient drug cost per discharge	−12.00 (−31.77 to 7.77); *P* = .225	69.46 (−3.84 to 142.75); *P* = .062	−164.53 (−199.80 to −129.26); *P* < .001	−176.53 (−205.31 to −147.74); *P* < .001	−753.18 (−924.76 to −581.60); *P* < .001
Outpatient drug cost share	−1.18 (−1.37 to −0.99); *P* < .001	−0.36 (−0.99 to 0.26); *P* = .246	0.41 (0.18 to 0.64); *P* = .001	−0.77 (−0.94 to −0.59); *P* < .001	1.70 (0.43 to 2.97); *P* = .010
Inpatient drug cost share	−2.03 (−2.20 to −1.87); *P* < .001	−0.15 (−0.78 to 0.47); *P* = .626	0.62 (0.40 to 0.83); *P* < .001	−1.42 (−1.55 to −1.28); *P* < .001	2.94 (1.51 to 4.36); *P* < .001

Values are regression coefficients (95% confidence intervals); *P* values. Models include province fixed effects and standard errors clustered by province (31 clusters; 434 province-year observations).

Cost outcomes are expressed in nominal CNY. Share outcomes are expressed in percentage points.

The difference in 2024 is the model-estimated policy-period trajectory minus continuation of the pre-2019 linear trend.

**Figure 2:**
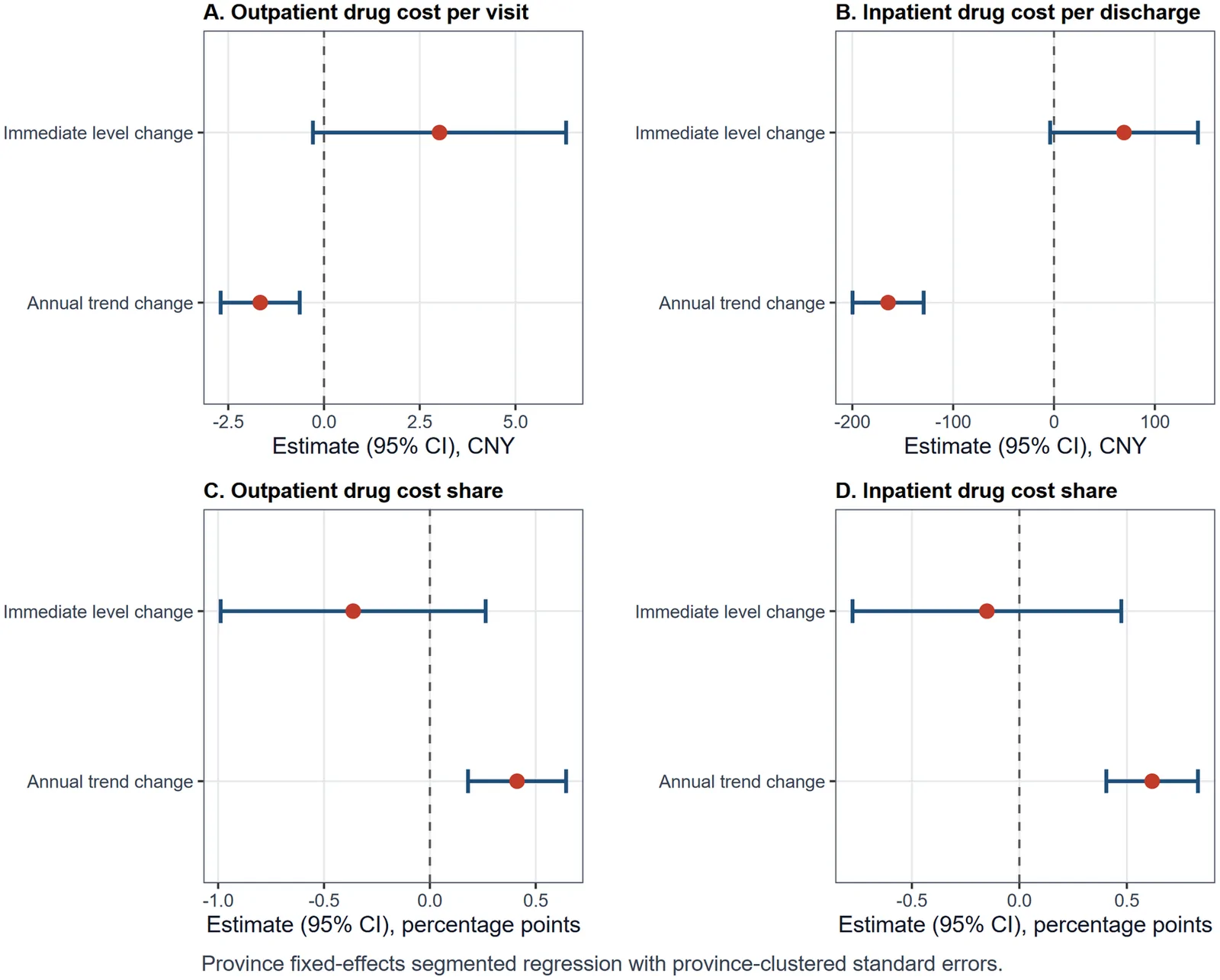
Immediate level and annual trend changes in public-hospital drug-cost burden at the 2019 breakpoint. Panels show (A) outpatient drug cost per visit, (B) inpatient drug cost per discharge, (C) outpatient drug-cost share, and (D) inpatient drug-cost share. In each panel, the upper estimate represents the immediate level change and the lower estimate represents the annual trend change. Points are coefficients and bars are 95% confidence intervals from province fixed-effects segmented regressions with standard errors clustered by province. Cost estimates are in CNY; share estimates are in percentage points.

Under the prespecified single-linear-pretrend model, the annual trend in outpatient drug cost per visit changed by −1.67 CNY per year after 2019 (95% CI, −2.70 to −0.63; *P* = .003), producing an estimated post-2019 net trend of 1.77 CNY per year (95% CI, 0.93 to 2.62). Importantly, the model-implied 2024 outpatient difference was −5.31 CNY and its interval crossed zero (95% CI, −11.70 to 1.07; *P* = .100). For inpatient drug cost per discharge, the linear-model trend changed by −164.53 CNY per year (95% CI, −199.80 to −129.26; *P* < .001), with a model-implied 2024 difference of −753.18 CNY (95% CI, −924.76 to −581.60; *P* < .001).

The annual trend changes in outpatient and inpatient drug-cost shares were positive: 0.41 (95% CI, 0.18–0.64; *P* = .001) and 0.62 (95% CI, 0.40–0.83; *P* < .001) percentage points per year, respectively. However, the post-2019 net trends remained negative at −0.77 (95% CI, −0.94 to −0.59) and −1.42 (95% CI, −1.55 to −1.28) percentage points per year. The positive trend-change coefficients therefore indicate that the shares continued to fall but less rapidly than their steep pre-2019 trajectories. Relative to continuation of the pre-2019 trends, the estimated 2024 differences were 1.70 percentage points (95% CI, 0.43–2.97; *P* = .010) for outpatient drug-cost share and 2.94 percentage points (95% CI, 1.51–4.36; *P* < .001) for inpatient drug-cost share.

### 3.3 Sensitivity and regional heterogeneity analyses

Within single-linear-pretrend analyses, the inpatient drug-cost trend-change estimate remained negative under alternative dependence structures and after omitting individual provinces or years. Leave-one-year-out estimates ranged from −237.69 to −99.10 CNY per year; after omitting 2024 the estimate was −99.10 CNY (*P* < .001). The outpatient range was −2.70 to −0.89 CNY per year, but omission of 2024 widened uncertainty across zero (−0.89 CNY; *P* = .105). CPI deflation produced trend changes of −1.33 CNY per outpatient visit (95% CI, −2.39 to −0.26; *P* = .016) and −122.31 CNY per inpatient discharge (95% CI, −157.87 to −86.75; *P* < .001).

The substantive conclusion was not robust to pretrend functional form. With a quadratic baseline trend, the outpatient and inpatient drug-cost trend changes became positive: 7.79 CNY (95% CI, 5.25 to 10.33) and 444.89 CNY (95% CI, 344.77 to 545.01) per year. With a piecewise pretrend allowing a distinct 2016–2018 slope, estimates were 1.64 CNY (95% CI, 0.27 to 3.02; *P* = .021) for outpatient cost and 17.11 CNY (95% CI, −33.46 to 67.68; *P* = .495) for inpatient cost. Share trend-change estimates remained positive under these specifications, indicating continued but slower post-2019 declines, although their magnitudes varied materially (Supplementary Table S10 and Supplementary Figure S5).

Non-drug costs changed at the same breakpoint. In the linear ITS, outpatient non-drug cost changed by −0.89 CNY per year (95% CI, −1.74 to −0.05; *P* = .039) and inpatient non-drug cost by −409.50 CNY per year (95% CI, −483.03 to −335.97; *P* < .001). In comparative models with province-by-component and full calendar-year fixed effects, the differential drug-versus-non-drug post-2019 trend changes were 1.58% per year for outpatient costs (95% CI, −0.08% to 3.27%; *P* = .060) and 1.03% per year for inpatient costs (95% CI, −2.06% to 4.22%; *P* = .488). Thus, the marked inpatient break was not drug-specific after common national year shocks were absorbed (Supplementary Table S11 and Supplementary Figure S6).

Benjamini-Hochberg correction did not change which primary trend-change tests met the.05 threshold under the linear model; FDR-adjusted q values were.003, <.001,.001, and <.001 for outpatient cost, inpatient cost, outpatient share, and inpatient share, respectively. Immediate-change q values ranged from .144 to .626. Breusch-Godfrey and Pesaran cross-sectional-dependence tests were significant for every primary model (all *P* < .001), supporting the use of alternative dependence-robust inference as sensitivity analyses (Supplementary Tables S13–S14).

Joint tests found no evidence that the annual trend changes differed among the 4 regions for outpatient drug cost (*P* = .725), inpatient drug cost (*P* = .305), outpatient drug-cost share (*P* = .550), or inpatient drug-cost share (*P* = .580) (Supplementary Tables S4–S5 and Supplementary Figure S2). Immediate level changes varied regionally for the 2 nominal cost outcomes, but these findings were exploratory and did not alter the overall absence of a clear national immediate change.

### 3.4 Exploratory revenue-structure analysis

The exact-scope revenue dataset contained 35 observations from 4 provinces selected only because suitable data were available. Drug income as a share of medical income declined from each province's earliest to latest observed year: by 10.64 percentage points in Chongqing (2016–2024), 5.24 points in Guangdong (2018–2024), 11.86 points in Gansu (2016–2022), and 15.55 points in Sichuan (2012–2023) (Table 3 and Figure 3). These unequal, data-availability-driven series are not representative of all provinces.

**Table 3:** Observed drug income as a share of medical income in public hospitals in 4 data-available Chinese provinces. Observed earliest-to-latest province-level changes.

Province	Years	Observations	Earliest value, %	Latest value, %	Absolute change, percentage points
Chongqing	2016–2024	9	38.58	27.94	−10.64
Guangdong	2018–2024	7	30.49	25.24	−5.24
Gansu	2016–2022	7	40.22	28.36	−11.86
Sichuan	2012–2023	12	39.62	24.07	−15.55

Provinces and observation periods were determined solely by availability of exact public-hospital-scope data; the trajectories are descriptive and not nationally representative.

Full segmented-model results, including wild cluster bootstrap-t inference, are reported in Supplementary Table S16.

**Figure 3:**
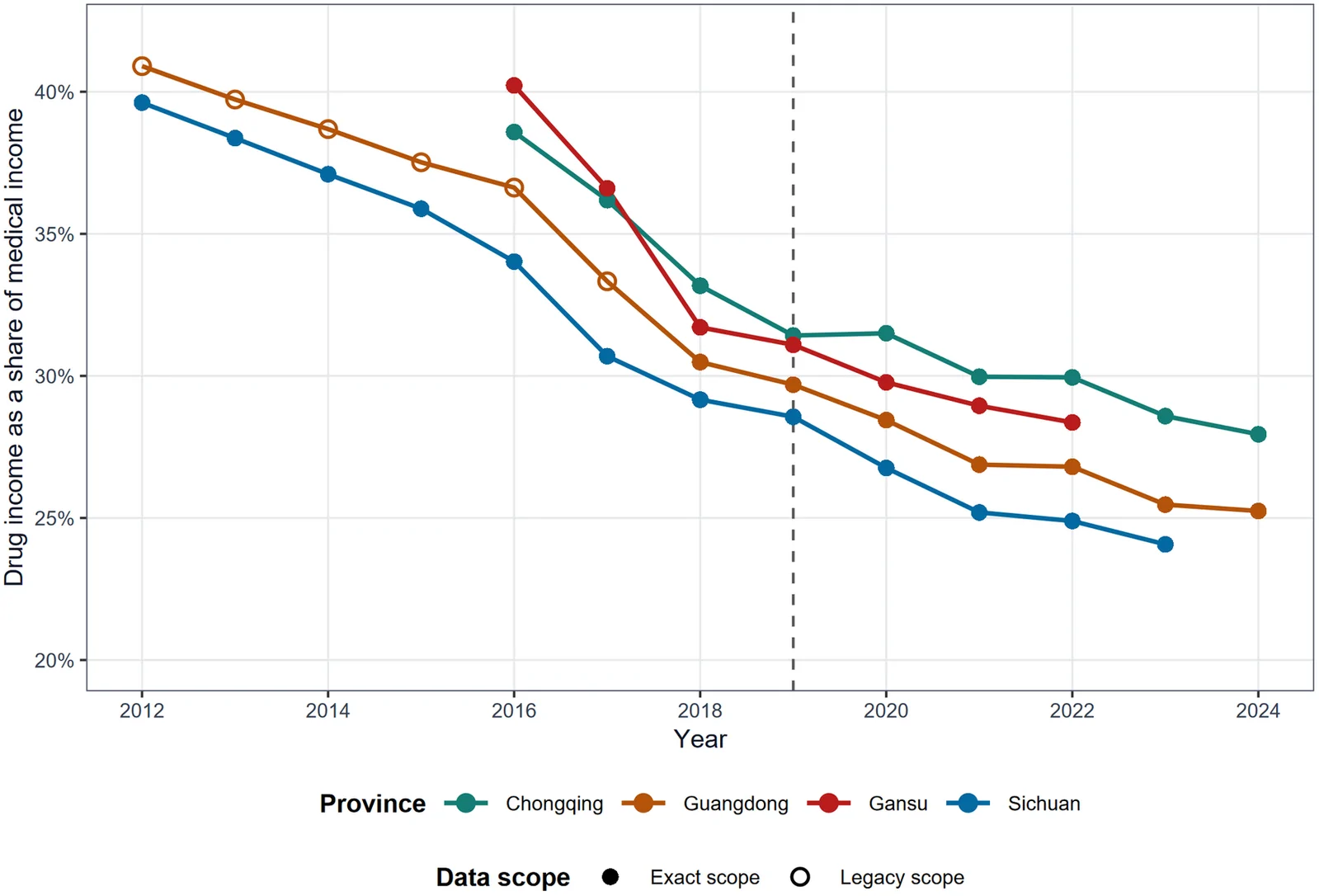
Exploratory trajectories of drug income as a share of medical income in public hospitals in 4 Chinese provinces. Filled points represent exact public-hospital statistical scope. Open points are Guangdong observations from 2012 through 2017 with a narrower legacy scope and were used only in sensitivity analysis. The vertical dashed line marks 2019. Province series have unequal observation periods.

Full inferential results were moved to the supplement. The conventional model estimated a trend change of 1.07 percentage points per year (95% CI, 0.16 to 1.99; *P* = .034), but the wild cluster bootstrap-t interval crossed zero (95% CI, −0.22 to 3.47; *P* = .055). The immediate-change bootstrap interval also crossed zero (*P* = .650). Together with only 4 clusters and the coincident accounting reform, these results support descriptive trajectory reporting only (Supplementary Table S16).

## 4 Discussion

### 4.1 Principal findings

In this 31-province study, the prespecified single-linear-pretrend model suggested downward changes in outpatient and inpatient drug-cost trends after 2019, without clear immediate level changes. However, the cost findings changed markedly when nonlinear or late-preperiod slopes were allowed, and a synchronous decline in non-drug inpatient costs weakened the specificity of the breakpoint to NVBP. Drug-cost shares remained substantially below their 2011 levels and continued to fall after 2019, but at slower rates than before 2019. The 4-province revenue series also declined descriptively, whereas inferential estimates were fragile and confounded by the accounting transition. In the prespecified linear model, the 2024 outpatient counterfactual difference was compatible with no difference, whereas the inpatient difference excluded zero; however, the inpatient result was neither robust to pretrend form nor drug-specific in the comparative model.

These findings distinguish 3 concepts that can otherwise be conflated. First, an immediate level change reflects a discontinuity in the intervention year; we found little evidence of such a jump. Second, a trend change compares post-2019 slope with the pre-2019 slope. A positive trend change in a share that was already decreasing means deceleration of the decline, not an absolute increase. Third, the counterfactual difference compares the estimated postpolicy series with indefinite continuation of the prepolicy linear trend. Positive 2024 differences in the cost shares therefore do not mean that drug shares increased; observed shares were substantially lower in 2024 than in 2011.

### 4.2 Comparison with previous studies and interpretation

City-level and product-level evaluations consistently show that NVBP can reduce prices and expenditures for selected medicines while altering use of winning, nonwinning, and alternative products (5–9). Their scope differs fundamentally from our all-medicine encounter-level outcomes. A literature-based reality check illustrates this dilution: the 25 molecules in the initial 4 + 7 pilot represented about 2% of hospital-channel sales in participating cities, although coverage expanded so that the first 6 batches involved purchasing amounts equal to about 30% of annual drug procurement by public medical institutions (10, 11). Therefore, a large aggregate 2019 trend break would be difficult to attribute exclusively to the earliest NVBP rounds, and the effect expected in an all-drug outcome should evolve as coverage expanded.

The study extends recent public-data panel work by isolating the drug component of outpatient and inpatient encounter costs and adding observations through 2024 (12). It also complements expenditure-decomposition evidence showing that service volume is an important driver of total public-hospital spending (13). A fall in drug cost per discharge should therefore not be interpreted as a proportional fall in total hospital drug expenditure, because total expenditure also depends on the number and composition of encounters.

The slower post-2019 decline in drug-cost shares admits at least 2 interpretations. A floor or saturation explanation is plausible: earlier zero-markup reform was associated nationally with 6.5% lower drug expenditure per visit and 9.5% lower drug expenditure per admission, and pharmaceutical revenue had already fallen from above 40% before 2009 to 31% in 2017 (1). Equally plausible is partial offsetting of selected-drug price savings by higher medical-service fees, greater use of nonselected or newly negotiated innovative medicines, diagnostic intensity, treatment intensity, or case-mix change. Because a share contains both a drug numerator and a total-cost denominator, growth in other services can also alter the ratio without a corresponding change in drug prices. The observed non-drug trajectories support this broader-system interpretation.

The revenue trajectories align descriptively with prior evidence that medicine-derived revenue fell after zero-markup and broader public-hospital reform (18–20). Nevertheless, the new government accounting system took effect on January 1, 2019, added and revised hospital accounting items and supplementary statements, and did not require prior-year comparative figures in the 2019 statements (14, 28). This creates a measurement discontinuity exactly at the ITS breakpoint that cannot be separated econometrically from NVBP. The 4-province revenue analysis therefore describes recorded series only and provides no credible estimate of an NVBP effect on revenue structure.

### 4.3 Policy implications

The results support continued monitoring of inpatient and outpatient drug costs after centralized procurement, but they do not establish that NVBP caused the observed aggregate break. Evaluation should jointly assess procurement prices, medicine volume, substitution to nonwinning and innovative products, treatment mix, non-drug services, total expenditure, and patient out-of-pocket payments. Higher-frequency procurement or hospital data with staggered exposure measures and credible comparison groups are needed to distinguish NVBP from payment, pricing, insurance-list, accounting, and pandemic-related changes.

Hospital financing also requires attention. Reducing medicine prices and drug-income dependence can improve affordability and weaken incentives tied to medicine sales, but hospitals require transparent compensation through appropriately priced professional services, strategic insurance payment, and clearly defined public subsidies (1, 2, 18–20). Stable accounting definitions and comparable public reporting are necessary to distinguish real changes in hospital financing from classification changes.

### 4.4 Strengths and limitations

This study used a balanced 14-year panel covering all 31 mainland province-level administrative units, separated outpatient and inpatient drug components, and evaluated both costs and structural shares. In response to the principal identification threats, it examined nonlinear and late-period pretrends, constructed non-drug control series, estimated comparative models with full calendar-year fixed effects, deflated costs, reported multiplicity-adjusted results and diagnostic statistics, and omitted individual years. All empirical results used source-backed observations; no simulated value was analyzed.

The study also has important limitations. First, the uncontrolled ITS has no contemporaneous untreated group. Full calendar-year fixed effects are collinear with the national intervention terms in the absolute model, so macroeconomic conditions and concurrent national reforms cannot be controlled directly. The comparative drug-versus-non-drug models absorb common year shocks but estimate relative component changes and do not solve confounding by policies that affect drug and non-drug components differently. Second, the single-linear counterfactual is poorly supported for some outcomes: quadratic and 2016–2018 piecewise specifications materially changed the cost estimates. Third, annual data provide only 8 prebreakpoint and 6 postbreakpoint observations and cannot represent exact implementation timing or rollout intensity. Fourth, province-level unweighted averages do not account for hospital size, patient volume, case mix, or within-province inequality. Fifth, CPI may not reflect health-specific price changes, and the outcomes do not measure total drug expenditure, reimbursement, or out-of-pocket burden. Sixth, diagnostics showed serial correlation and strong cross-sectional dependence despite alternative inference checks. Seventh, yearbook definitions and revisions may differ across years. Finally, the revenue analysis contains only 35 observations from 4 availability-selected provinces, has 4 clusters and unequal periods, and is structurally confounded by the 2019 accounting reform.

## 5 Conclusions

Across 31 Chinese provinces, a single-linear-pretrend ITS suggested changes in public-hospital drug-cost trajectories after 2019, but these estimates were highly sensitive to pretrend specification and coincided with substantial changes in non-drug costs. Drug-cost shares remained far below 2011 levels and continued to decline, although more slowly. The available 4-province revenue data are descriptive only. Annual aggregate data cannot identify an NVBP-specific effect amid concurrent payment, pricing, insurance-list, accounting, and pandemic-related changes; stronger causal evaluation requires higher-frequency, exposure-specific data and credible contemporaneous comparisons.

## Data Availability

The data analyzed in this study were obtained from publicly available national and provincial statistical yearbooks, as described in the Methods. Derived results and supporting analyses are provided in the article and Supplementary Material. Further inquiries may be directed to the corresponding author.
