# Supplementary material for: Drug-cost trajectories after national volume-based procurement in Chinese public hospitals: a 31-province interrupted time-series analysis
Source: Front Med (Lausanne). 2026 Sep 30;13:1970115. doi: 10.3389/fmed.2026.1970115 (PMC13661909; doi:10.3389/fmed.2026.1970115)
Supplement: Supplementary file 1 [file Datasheet1.docx]

Supplementary Material

# 1 Supplementary tables

**Supplementary Table S1. Sensitivity analyses of annual trend changes in drug-cost burden**

| Model | Outcome | Annual trend change (95% confidence interval) | P value | Direction matches primary | Observations |
| --- | --- | --- | --- | --- | --- |
| Primary: province-clustered SE | Outpatient drug cost per visit | -1.67 (-2.70, -0.63) | .003 | Yes | 434 |
| Primary: province-clustered SE | Inpatient drug cost per discharge | -164.53 (-199.80, -129.26) | <.001 | Yes | 434 |
| Primary: province-clustered SE | Outpatient drug cost share | 0.41 (0.18, 0.64) | .001 | Yes | 434 |
| Primary: province-clustered SE | Inpatient drug cost share | 0.62 (0.40, 0.83) | <.001 | Yes | 434 |
| Alternative 2020 breakpoint | Outpatient drug cost per visit | -2.67 (-3.90, -1.45) | <.001 | Yes | 434 |
| COVID-period indicator | Outpatient drug cost per visit | -1.37 (-2.42, -0.32) | .013 | Yes | 434 |
| Exclude 2020-2022 | Outpatient drug cost per visit | -1.20 (-2.39, -0.01) | .048 | Yes | 341 |
| Province-specific trends | Outpatient drug cost per visit | -1.67 (-2.74, -0.60) | .003 | Yes | 434 |
| Two-way clustered SE | Outpatient drug cost per visit | -1.67 (-3.69, 0.36) | .103 | Yes | 434 |
| Alternative 2020 breakpoint | Inpatient drug cost per discharge | -226.06 (-270.97, -181.16) | <.001 | Yes | 434 |
| COVID-period indicator | Inpatient drug cost per discharge | -136.08 (-168.77, -103.40) | <.001 | Yes | 434 |
| Exclude 2020-2022 | Inpatient drug cost per discharge | -142.93 (-177.34, -108.53) | <.001 | Yes | 341 |
| Province-specific trends | Inpatient drug cost per discharge | -164.53 (-201.10, -127.96) | <.001 | Yes | 434 |
| Two-way clustered SE | Inpatient drug cost per discharge | -164.53 (-286.23, -42.82) | .010 | Yes | 434 |
| Alternative 2020 breakpoint | Outpatient drug cost share | 0.59 (0.34, 0.84) | <.001 | Yes | 434 |
| COVID-period indicator | Outpatient drug cost share | 0.36 (0.13, 0.59) | .003 | Yes | 434 |
| Exclude 2020-2022 | Outpatient drug cost share | 0.31 (0.06, 0.55) | .016 | Yes | 341 |
| Province-specific trends | Outpatient drug cost share | 0.41 (0.17, 0.65) | .001 | Yes | 434 |
| Two-way clustered SE | Outpatient drug cost share | 0.41 (0.04, 0.78) | .032 | Yes | 434 |
| Alternative 2020 breakpoint | Inpatient drug cost share | 0.55 (0.32, 0.78) | <.001 | Yes | 434 |
| COVID-period indicator | Inpatient drug cost share | 0.66 (0.46, 0.87) | <.001 | Yes | 434 |
| Exclude 2020-2022 | Inpatient drug cost share | 0.60 (0.39, 0.81) | <.001 | Yes | 341 |
| Province-specific trends | Inpatient drug cost share | 0.62 (0.40, 0.84) | <.001 | Yes | 434 |
| Two-way clustered SE | Inpatient drug cost share | 0.62 (0.07, 1.17) | .029 | Yes | 434 |
| Annual-mean Newey-West | Outpatient drug cost per visit | -1.67 (-4.06, 0.73) | .153 | Yes | 14 |
| Annual-mean Newey-West | Inpatient drug cost per discharge | -164.53 (-330.37, 1.31) | .052 | Yes | 14 |
| Annual-mean Newey-West | Outpatient drug cost share | 0.41 (0.01, 0.82) | .046 | Yes | 14 |
| Annual-mean Newey-West | Inpatient drug cost share | 0.62 (-0.12, 1.35) | .091 | Yes | 14 |
| Driscoll-Kraay SE | Outpatient drug cost per visit | -1.67 (-3.64, 0.31) | .091 | Yes | 434 |
| Driscoll-Kraay SE | Inpatient drug cost per discharge | -164.53 (-300.90, -28.16) | .022 | Yes | 434 |
| Driscoll-Kraay SE | Outpatient drug cost share | 0.41 (0.08, 0.74) | .019 | Yes | 434 |
| Driscoll-Kraay SE | Inpatient drug cost share | 0.62 (0.01, 1.22) | .046 | Yes | 434 |

Note. Cost estimates are in Chinese yuan; share estimates are in percentage points.

All models use observed yearbook data. No simulated value was included.

**Supplementary Table S2. Log-linear sensitivity analyses of nominal drug costs**

| Outcome | Annual trend change, percent (95% confidence interval) | P value | Observations |
| --- | --- | --- | --- |
| Outpatient drug cost per visit | -2.34 (-3.33, -1.33) | <.001 | 434 |
| Inpatient drug cost per discharge | -6.74 (-8.02, -5.45) | <.001 | 434 |

Note. Values are approximate annual percentage changes derived from log-outcome segmented models.

**Supplementary Table S3. Leave-one-province-out analysis of drug-cost burden**

| Outcome | Full-sample immediate estimate | Range after omitting 1 province | Immediate direction stable | Full-sample trend estimate | Trend range after omitting 1 province | Trend direction stable |
| --- | --- | --- | --- | --- | --- | --- |
| Inpatient drug cost per discharge | 69.46 | 57.41 to 84.31 | Yes | -164.53 | -170.56 to -158.12 | Yes |
| Inpatient drug cost share | -0.15 | -0.29 to -0.05 | Yes | 0.62 | 0.57 to 0.65 | Yes |
| Outpatient drug cost per visit | 3.02 | 2.09 to 3.57 | Yes | -1.67 | -1.88 to -1.47 | Yes |
| Outpatient drug cost share | -0.36 | -0.48 to -0.25 | Yes | 0.41 | 0.37 to 0.46 | Yes |

Note. Cost estimates are in Chinese yuan; share estimates are in percentage points.

**Supplementary Table S4. Exploratory region-specific segmented regression estimates**

| Region | Provinces | Outcome | Effect | Estimate (95% confidence interval) | P value |
| --- | --- | --- | --- | --- | --- |
| Northeastern | 3 | Outpatient drug cost per visit | Immediate level change | 4.14 (-2.14, 10.43) | .188 |
| Northeastern | 3 | Outpatient drug cost per visit | Annual trend change | -1.92 (-5.26, 1.43) | .251 |
| Northeastern | 3 | Inpatient drug cost per discharge | Immediate level change | 489.09 (261.82, 716.36) | <.001 |
| Northeastern | 3 | Inpatient drug cost per discharge | Annual trend change | -248.25 (-347.86, -148.65) | <.001 |
| Northeastern | 3 | Outpatient drug cost share | Immediate level change | -1.20 (-2.43, 0.03) | .055 |
| Northeastern | 3 | Outpatient drug cost share | Annual trend change | 0.26 (-0.59, 1.11) | .539 |
| Northeastern | 3 | Inpatient drug cost share | Immediate level change | -1.38 (-2.73, -0.03) | .045 |
| Northeastern | 3 | Inpatient drug cost share | Annual trend change | 0.29 (-0.21, 0.79) | .243 |
| Eastern | 10 | Outpatient drug cost per visit | Immediate level change | 10.55 (4.03, 17.07) | .002 |
| Eastern | 10 | Outpatient drug cost per visit | Annual trend change | -0.92 (-2.91, 1.08) | .354 |
| Eastern | 10 | Inpatient drug cost per discharge | Immediate level change | 337.29 (209.39, 465.18) | <.001 |
| Eastern | 10 | Inpatient drug cost per discharge | Annual trend change | -163.71 (-233.59, -93.83) | <.001 |
| Eastern | 10 | Outpatient drug cost share | Immediate level change | -0.20 (-1.46, 1.06) | .749 |
| Eastern | 10 | Outpatient drug cost share | Annual trend change | 0.57 (0.21, 0.92) | .003 |
| Eastern | 10 | Inpatient drug cost share | Immediate level change | 0.01 (-0.74, 0.75) | .986 |
| Eastern | 10 | Inpatient drug cost share | Annual trend change | 0.71 (0.26, 1.16) | .003 |
| Western | 12 | Outpatient drug cost per visit | Immediate level change | -0.97 (-4.74, 2.80) | .602 |
| Western | 12 | Outpatient drug cost per visit | Annual trend change | -1.89 (-3.61, -0.18) | .031 |
| Western | 12 | Inpatient drug cost per discharge | Immediate level change | 52.79 (-74.39, 179.96) | .403 |
| Western | 12 | Inpatient drug cost per discharge | Annual trend change | -141.79 (-193.06, -90.52) | <.001 |
| Western | 12 | Outpatient drug cost share | Immediate level change | -1.44 (-2.44, -0.45) | .006 |
| Western | 12 | Outpatient drug cost share | Annual trend change | 0.41 (-0.03, 0.85) | .067 |
| Western | 12 | Inpatient drug cost share | Immediate level change | -1.43 (-2.47, -0.40) | .008 |
| Western | 12 | Inpatient drug cost share | Annual trend change | 0.58 (0.32, 0.83) | <.001 |
| Central | 6 | Outpatient drug cost per visit | Immediate level change | 6.48 (0.78, 12.18) | .027 |
| Central | 6 | Outpatient drug cost per visit | Annual trend change | -2.33 (-3.86, -0.80) | .004 |
| Central | 6 | Inpatient drug cost per discharge | Immediate level change | 296.66 (212.25, 381.07) | <.001 |
| Central | 6 | Inpatient drug cost per discharge | Annual trend change | -169.50 (-232.66, -106.35) | <.001 |
| Central | 6 | Outpatient drug cost share | Immediate level change | -0.18 (-1.82, 1.46) | .824 |
| Central | 6 | Outpatient drug cost share | Annual trend change | 0.24 (-0.07, 0.54) | .123 |
| Central | 6 | Inpatient drug cost share | Immediate level change | -0.43 (-1.56, 0.70) | .443 |
| Central | 6 | Inpatient drug cost share | Annual trend change | 0.71 (0.18, 1.25) | .011 |

Note. Cost estimates are in Chinese yuan; share estimates are in percentage points.

Regional estimates came from a common interaction model. Northeastern China contained only 3 provinces.

**Supplementary Table S5. Joint tests of regional heterogeneity**

| Outcome | Effect | F statistic | Numerator degrees of freedom | Denominator degrees of freedom | P value |
| --- | --- | --- | --- | --- | --- |
| Outpatient drug cost per visit | Immediate level change | 3.96 | 3 | 30 | .017 |
| Outpatient drug cost per visit | Annual trend change | 0.44 | 3 | 30 | .725 |
| Inpatient drug cost per discharge | Immediate level change | 5.86 | 3 | 30 | .003 |
| Inpatient drug cost per discharge | Annual trend change | 1.26 | 3 | 30 | .305 |
| Outpatient drug cost share | Immediate level change | 1.20 | 3 | 30 | .327 |
| Outpatient drug cost share | Annual trend change | 0.72 | 3 | 30 | .550 |
| Inpatient drug cost share | Immediate level change | 2.32 | 3 | 30 | .095 |
| Inpatient drug cost share | Annual trend change | 0.67 | 3 | 30 | .580 |

Note. Joint Wald tests evaluated equality of region-specific coefficients.

**Supplementary Table S6. Sensitivity analyses of exploratory drug-income dependence**

| Specification | Method | Effect | Estimate (95% confidence interval), percentage points | P value | Observations | Provinces |
| --- | --- | --- | --- | --- | --- | --- |
| Primary exact-scope unbalanced | Province-clustered HC1 | Annual trend change | 1.07 (0.16, 1.99) | .034 | 35 | 4 |
| Primary exact-scope unbalanced | Province-clustered HC1 | Post-policy annual trend | -0.85 (-1.10, -0.60) | .002 | 35 | 4 |
| Primary exact-scope unbalanced | Province-clustered HC1 | Model-implied difference in 2022 | 3.04 (-0.11, 6.19) | .055 | 35 | 4 |
| Balanced CQ-GS-SC 2016-2022 | Province-clustered HC1 | Annual trend change | 2.21 (-0.93, 5.35) | .094 | 21 | 3 |
| Balanced CQ-GS-SC 2016-2022 | Province-clustered HC1 | Post-policy annual trend | -0.92 (-1.87, 0.03) | .053 | 21 | 3 |
| Balanced CQ-GS-SC 2016-2022 | Province-clustered HC1 | Model-implied difference in 2022 | 8.66 (-3.23, 20.55) | .088 | 21 | 3 |
| All-source scope sensitivity | Province-clustered HC1 | Annual trend change | 0.93 (0.40, 1.45) | .011 | 41 | 4 |
| All-source scope sensitivity | Province-clustered HC1 | Post-policy annual trend | -0.85 (-1.12, -0.58) | .002 | 41 | 4 |
| All-source scope sensitivity | Province-clustered HC1 | Model-implied difference in 2022 | 2.16 (0.43, 3.89) | .029 | 41 | 4 |
| Exact scope plus COVID-period indicator | Province-clustered HC1 | Annual trend change | 1.06 (0.11, 2.02) | .038 | 35 | 4 |
| Exact scope plus COVID-period indicator | Province-clustered HC1 | Post-policy annual trend | -0.86 (-1.14, -0.58) | .002 | 35 | 4 |
| Exact scope plus COVID-period indicator | Province-clustered HC1 | Model-implied difference in 2022 | 3.21 (0.33, 6.08) | .038 | 35 | 4 |
| Exact scope alternative 2020 breakpoint | Province-clustered HC1 | Annual trend change | 1.16 (0.35, 1.96) | .020 | 35 | 4 |
| Exact scope alternative 2020 breakpoint | Province-clustered HC1 | Post-policy annual trend | -0.76 (-0.94, -0.58) | <.001 | 35 | 4 |
| Exact scope alternative 2020 breakpoint | Province-clustered HC1 | Model-implied difference in 2022 | 2.95 (-0.22, 6.13) | .060 | 35 | 4 |
| Primary exact-scope unbalanced | GLS AR(1), model-based sensitivity | Annual trend change | 1.55 (0.84, 2.25) | <.001 | 35 | 4 |
| Primary exact-scope unbalanced | GLS AR(1), model-based sensitivity | Post-policy annual trend | -0.89 (-1.32, -0.46) | <.001 | 35 | 4 |
| Primary exact-scope unbalanced | GLS AR(1), model-based sensitivity | Model-implied difference in 2022 | 6.14 (3.39, 8.88) | <.001 | 35 | 4 |

Note. Cluster-robust analyses used only 3 or 4 province clusters and are nonconfirmatory.

The generalized least-squares autoregressive model is a model-based sensitivity analysis; its estimated correlation parameter was close to the boundary.

**Supplementary Table S7. Province-level heterogeneity in exploratory drug-income dependence**

| Province | Years | Observations | Earliest value, % | Latest value, % | Observed change, percentage points | Province-specific trend change (95% confidence interval) |
| --- | --- | --- | --- | --- | --- | --- |
| Chongqing | 2016-2024 | 9 | 38.58 | 27.94 | -10.64 | Not estimable |
| Guangdong | 2018-2024 | 7 | 30.49 | 25.24 | -5.24 | 0.85 (-0.39, 2.09) |
| Gansu | 2016-2022 | 7 | 40.22 | 28.36 | -11.86 | Not estimable |
| Sichuan | 2012-2023 | 12 | 39.62 | 24.07 | -15.55 | 0.62 (-0.53, 1.76) |

Note. Province-specific segmented models were fitted only where the available preperiod and postperiod observations permitted estimation.

**Supplementary Table S8. Leave-one-province-out analysis of exploratory drug-income dependence**

| Effect | Minimum estimate, percentage points | Maximum estimate, percentage points | Direction stable | Minimum confidence limit | Maximum confidence limit | Models |
| --- | --- | --- | --- | --- | --- | --- |
| Annual trend change | 1.00 | 2.67 | Yes | 0.51 | 3.44 | 4 |
| Model-implied difference in 2022 | 3.72 | 10.39 | Yes | 1.78 | 13.51 | 4 |

Note. Intervals used province-clustered standard errors with only 3 remaining clusters and should be interpreted cautiously.

**Supplementary Table S9. Exploratory secondary hospital revenue and expenditure outcomes**

| Outcome | Effect | Estimate (95% confidence interval), percentage points | P value | Observations | Provinces |
| --- | --- | --- | --- | --- | --- |
| Fiscal income share of total income | Immediate 2019 change | 3.31 (1.30 to 5.33) | .014 | 31 | 4 |
| Fiscal income share of total income | Annual trend change | 0.21 (-1.56 to 1.99) | .729 | 31 | 4 |
| Fiscal income share of total income | Post-2019 annual trend | 0.33 (-1.20 to 1.85) | .543 | 31 | 4 |
| Fiscal income share of total income | Difference in 2022 | 3.95 (-0.24 to 8.13) | .058 | 31 | 4 |
| Medical income share of total income | Immediate 2019 change | -3.14 (-4.91 to -1.37) | .011 | 35 | 4 |
| Medical income share of total income | Annual trend change | -0.32 (-1.90 to 1.25) | .559 | 35 | 4 |
| Medical income share of total income | Post-2019 annual trend | -0.51 (-1.87 to 0.86) | .322 | 35 | 4 |
| Medical income share of total income | Difference in 2022 | -4.11 (-7.55 to -0.67) | .032 | 35 | 4 |
| Personnel expense share of total expense | Immediate 2019 change | -0.03 (-2.55 to 2.50) | .976 | 31 | 4 |
| Personnel expense share of total expense | Annual trend change | -0.74 (-1.25 to -0.23) | .019 | 31 | 4 |
| Personnel expense share of total expense | Post-2019 annual trend | 0.51 (0.23 to 0.80) | .010 | 31 | 4 |
| Personnel expense share of total expense | Difference in 2022 | -2.25 (-5.84 to 1.34) | .140 | 31 | 4 |
| Drug expense share of total expense | Immediate 2019 change | -0.34 (-1.00 to 0.33) | .163 | 28 | 3 |
| Drug expense share of total expense | Annual trend change | 0.19 (-1.20 to 1.59) | .614 | 28 | 3 |
| Drug expense share of total expense | Post-2019 annual trend | -1.01 (-2.09 to 0.06) | .055 | 28 | 3 |
| Drug expense share of total expense | Difference in 2022 | 0.24 (-4.53 to 5.01) | .849 | 28 | 3 |
| Operating balance ratio | Immediate 2019 change | 5.65 (2.34 to 8.97) | .012 | 35 | 4 |
| Operating balance ratio | Annual trend change | 0.32 (-1.48 to 2.12) | .610 | 35 | 4 |
| Operating balance ratio | Post-2019 annual trend | -0.37 (-1.81 to 1.08) | .478 | 35 | 4 |
| Operating balance ratio | Difference in 2022 | 6.62 (2.08 to 11.16) | .019 | 35 | 4 |

Note. Outcome-specific analyses used all available exact-scope observations without imputation.

All estimates are exploratory because only 3 or 4 province clusters contributed to each model.

**Supplementary Table S10. Sensitivity of annual trend-change estimates to pretrend functional form**

| Outcome | Pretrend specification | Estimate (95% CI) | P value | Unit |
| --- | --- | --- | --- | --- |
| Outpatient drug cost per visit | Single linear pretrend, 2011-2018 | -1.67 (-2.70 to -0.63) | .003 | CNY/year |
| Outpatient drug cost per visit | Quadratic baseline trend | 7.79 (5.25 to 10.33) | <.001 | CNY/year |
| Outpatient drug cost per visit | Piecewise pretrend, 2016-2018 segment | 1.64 (0.27 to 3.02) | .021 | CNY/year |
| Inpatient drug cost per discharge | Single linear pretrend, 2011-2018 | -164.53 (-199.80 to -129.26) | <.001 | CNY/year |
| Inpatient drug cost per discharge | Quadratic baseline trend | 444.89 (344.77 to 545.01) | <.001 | CNY/year |
| Inpatient drug cost per discharge | Piecewise pretrend, 2016-2018 segment | 17.11 (-33.46 to 67.68) | .495 | CNY/year |
| Outpatient drug-cost share | Single linear pretrend, 2011-2018 | 0.41 (0.18 to 0.64) | .001 | percentage points/year |
| Outpatient drug-cost share | Quadratic baseline trend | 1.93 (1.19 to 2.66) | <.001 | percentage points/year |
| Outpatient drug-cost share | Piecewise pretrend, 2016-2018 segment | 1.18 (0.77 to 1.59) | <.001 | percentage points/year |
| Inpatient drug-cost share | Single linear pretrend, 2011-2018 | 0.62 (0.40 to 0.83) | <.001 | percentage points/year |
| Inpatient drug-cost share | Quadratic baseline trend | 4.09 (3.47 to 4.71) | <.001 | percentage points/year |
| Inpatient drug-cost share | Piecewise pretrend, 2016-2018 segment | 1.92 (1.62 to 2.22) | <.001 | percentage points/year |

Note. All models include province fixed effects and use province-clustered standard errors. The piecewise specification allows a distinct 2016-2018 preintervention slope.

**Supplementary Table S11. Non-drug control series and drug-versus-non-drug specificity analyses**

| Outcome/comparison | Model | Trend-change estimate (95% CI) | P value | Unit |
| --- | --- | --- | --- | --- |
| Outpatient non-drug cost per visit | Non-drug control-series ITS | -0.89 (-1.74 to -0.05) | .039 | CNY/year |
| Inpatient non-drug cost per discharge | Non-drug control-series ITS | -409.50 (-483.03 to -335.97) | <.001 | CNY/year |
| Outpatient drug vs non-drug | Comparative model with full year fixed effects | 1.58 (-0.08 to 3.27) | .060 | Approx. %/year |
| Inpatient drug vs non-drug | Comparative model with full year fixed effects | 1.03 (-2.06 to 4.22) | .488 | Approx. %/year |

Note. Non-drug cost equals total medical cost minus drug cost. Comparative models include province-by-component and full calendar-year fixed effects with 2-way province-and-year clustered standard errors. For the comparative models, coefficients and confidence limits were transformed as [exp(β) − 1] × 100 and are reported as percentage changes.

**Supplementary Table S12. CPI-deflated sensitivity analysis of drug costs**

| Outcome | Price basis | Trend change (95% CI), CNY/year | P value | Observations |
| --- | --- | --- | --- | --- |
| Outpatient drug cost per visit | Nominal CNY | -1.67 (-2.70 to -0.63) | .003 | 434 |
| Outpatient drug cost per visit, 2019 CNY | CPI-deflated, 2019 CNY | -1.33 (-2.39 to -0.26) | .016 | 434 |
| Inpatient drug cost per discharge | Nominal CNY | -164.53 (-199.80 to -129.26) | <.001 | 434 |
| Inpatient drug cost per discharge, 2019 CNY | CPI-deflated, 2019 CNY | -122.31 (-157.87 to -86.75) | <.001 | 434 |

Note. Constant-price outcomes use China's annual CPI (2010 = 100) and are expressed in 2019 CNY.

**Supplementary Table S13. Benjamini-Hochberg false-discovery-rate correction for primary linear ITS tests**

| Outcome | Effect family | Estimate | Raw P | BH q | Unit |
| --- | --- | --- | --- | --- | --- |
| Outpatient drug cost per visit | Immediate 2019 level change | 3.02 | .072 | .144 | CNY |
| Outpatient drug cost per visit | Annual trend change after 2019 | -1.67 | .003 | .003 | CNY |
| Inpatient drug cost per discharge | Immediate 2019 level change | 69.46 | .062 | .144 | CNY |
| Inpatient drug cost per discharge | Annual trend change after 2019 | -164.53 | <.001 | <.001 | CNY |
| Outpatient drug-cost share | Immediate 2019 level change | -0.36 | .246 | .329 | percentage points |
| Outpatient drug-cost share | Annual trend change after 2019 | 0.41 | .001 | .001 | percentage points |
| Inpatient drug-cost share | Immediate 2019 level change | -0.15 | .626 | .626 | percentage points |
| Inpatient drug-cost share | Annual trend change after 2019 | 0.62 | <.001 | <.001 | percentage points |

Note. Corrections were performed separately for the immediate-change family and annual-trend-change family across the 4 primary outcomes.

**Supplementary Table S14. Residual diagnostic tests for primary linear ITS models**

| Outcome | Breusch-Godfrey statistic | P value | Pesaran CD statistic | P value |
| --- | --- | --- | --- | --- |
| Outpatient drug cost per visit | 142.365 | <.001 | 14.358 | <.001 |
| Inpatient drug cost per discharge | 183.498 | <.001 | 33.167 | <.001 |
| Outpatient drug-cost share | 149.066 | <.001 | 13.819 | <.001 |
| Inpatient drug-cost share | 161.460 | <.001 | 24.535 | <.001 |

Note. Significant tests indicate residual serial correlation or cross-sectional dependence, respectively.

**Supplementary Table S15. Leave-one-year-out analysis of annual trend changes**

| Outcome | Omitted year | Estimate (95% CI) | P value | Unit |
| --- | --- | --- | --- | --- |
| Outpatient drug cost per visit | 2011 | -1.05 (-2.13 to 0.03) | .056 | CNY/year |
| Outpatient drug cost per visit | 2012 | -1.56 (-2.58 to -0.54) | .004 | CNY/year |
| Outpatient drug cost per visit | 2013 | -1.73 (-2.76 to -0.70) | .002 | CNY/year |
| Outpatient drug cost per visit | 2014 | -1.72 (-2.75 to -0.68) | .002 | CNY/year |
| Outpatient drug cost per visit | 2015 | -1.60 (-2.64 to -0.57) | .004 | CNY/year |
| Outpatient drug cost per visit | 2016 | -1.56 (-2.59 to -0.52) | .004 | CNY/year |
| Outpatient drug cost per visit | 2017 | -1.87 (-2.90 to -0.85) | <.001 | CNY/year |
| Outpatient drug cost per visit | 2018 | -2.24 (-3.28 to -1.20) | <.001 | CNY/year |
| Outpatient drug cost per visit | 2019 | -2.70 (-3.93 to -1.47) | <.001 | CNY/year |
| Outpatient drug cost per visit | 2020 | -1.08 (-2.26 to 0.10) | .071 | CNY/year |
| Outpatient drug cost per visit | 2021 | -1.77 (-2.79 to -0.75) | .001 | CNY/year |
| Outpatient drug cost per visit | 2022 | -1.77 (-2.83 to -0.71) | .002 | CNY/year |
| Outpatient drug cost per visit | 2023 | -1.82 (-2.81 to -0.84) | <.001 | CNY/year |
| Outpatient drug cost per visit | 2024 | -0.89 (-1.99 to 0.20) | .105 | CNY/year |
| Inpatient drug cost per discharge | 2011 | -132.57 (-168.72 to -96.41) | <.001 | CNY/year |
| Inpatient drug cost per discharge | 2012 | -159.12 (-194.51 to -123.73) | <.001 | CNY/year |
| Inpatient drug cost per discharge | 2013 | -167.76 (-203.12 to -132.41) | <.001 | CNY/year |
| Inpatient drug cost per discharge | 2014 | -166.51 (-201.69 to -131.33) | <.001 | CNY/year |
| Inpatient drug cost per discharge | 2015 | -161.26 (-196.62 to -125.89) | <.001 | CNY/year |
| Inpatient drug cost per discharge | 2016 | -156.04 (-190.85 to -121.22) | <.001 | CNY/year |
| Inpatient drug cost per discharge | 2017 | -172.40 (-207.97 to -136.83) | <.001 | CNY/year |
| Inpatient drug cost per discharge | 2018 | -202.73 (-239.22 to -166.25) | <.001 | CNY/year |
| Inpatient drug cost per discharge | 2019 | -237.69 (-282.95 to -192.43) | <.001 | CNY/year |
| Inpatient drug cost per discharge | 2020 | -155.60 (-190.58 to -120.63) | <.001 | CNY/year |
| Inpatient drug cost per discharge | 2021 | -159.10 (-193.89 to -124.31) | <.001 | CNY/year |
| Inpatient drug cost per discharge | 2022 | -172.42 (-208.85 to -135.99) | <.001 | CNY/year |
| Inpatient drug cost per discharge | 2023 | -165.37 (-199.18 to -131.55) | <.001 | CNY/year |
| Inpatient drug cost per discharge | 2024 | -99.10 (-133.03 to -65.17) | <.001 | CNY/year |
| Outpatient drug-cost share | 2011 | 0.57 (0.32 to 0.83) | <.001 | percentage points/year |
| Outpatient drug-cost share | 2012 | 0.41 (0.18 to 0.65) | .001 | percentage points/year |
| Outpatient drug-cost share | 2013 | 0.41 (0.18 to 0.64) | <.001 | percentage points/year |
| Outpatient drug-cost share | 2014 | 0.40 (0.17 to 0.63) | .001 | percentage points/year |
| Outpatient drug-cost share | 2015 | 0.42 (0.19 to 0.66) | <.001 | percentage points/year |
| Outpatient drug-cost share | 2016 | 0.45 (0.21 to 0.68) | <.001 | percentage points/year |
| Outpatient drug-cost share | 2017 | 0.36 (0.13 to 0.59) | .003 | percentage points/year |
| Outpatient drug-cost share | 2018 | 0.27 (0.05 to 0.49) | .017 | percentage points/year |
| Outpatient drug-cost share | 2019 | 0.61 (0.37 to 0.85) | <.001 | percentage points/year |
| Outpatient drug-cost share | 2020 | 0.39 (0.14 to 0.63) | .003 | percentage points/year |
| Outpatient drug-cost share | 2021 | 0.38 (0.14 to 0.61) | .002 | percentage points/year |
| Outpatient drug-cost share | 2022 | 0.40 (0.16 to 0.63) | .002 | percentage points/year |
| Outpatient drug-cost share | 2023 | 0.43 (0.21 to 0.66) | <.001 | percentage points/year |
| Outpatient drug-cost share | 2024 | 0.31 (0.07 to 0.55) | .013 | percentage points/year |
| Inpatient drug-cost share | 2011 | 0.84 (0.62 to 1.06) | <.001 | percentage points/year |
| Inpatient drug-cost share | 2012 | 0.64 (0.43 to 0.86) | <.001 | percentage points/year |
| Inpatient drug-cost share | 2013 | 0.61 (0.40 to 0.82) | <.001 | percentage points/year |
| Inpatient drug-cost share | 2014 | 0.60 (0.39 to 0.81) | <.001 | percentage points/year |
| Inpatient drug-cost share | 2015 | 0.64 (0.43 to 0.85) | <.001 | percentage points/year |
| Inpatient drug-cost share | 2016 | 0.68 (0.47 to 0.89) | <.001 | percentage points/year |
| Inpatient drug-cost share | 2017 | 0.56 (0.36 to 0.76) | <.001 | percentage points/year |
| Inpatient drug-cost share | 2018 | 0.35 (0.10 to 0.59) | .008 | percentage points/year |
| Inpatient drug-cost share | 2019 | 0.52 (0.27 to 0.77) | <.001 | percentage points/year |
| Inpatient drug-cost share | 2020 | 0.60 (0.39 to 0.81) | <.001 | percentage points/year |
| Inpatient drug-cost share | 2021 | 0.62 (0.41 to 0.83) | <.001 | percentage points/year |
| Inpatient drug-cost share | 2022 | 0.59 (0.37 to 0.81) | <.001 | percentage points/year |
| Inpatient drug-cost share | 2023 | 0.57 (0.36 to 0.78) | <.001 | percentage points/year |
| Inpatient drug-cost share | 2024 | 0.86 (0.67 to 1.06) | <.001 | percentage points/year |

Note. Each row refits the primary single-linear-pretrend model after omitting all 31 observations from the indicated calendar year.

**Supplementary Table S16. Full exploratory model for drug income as a share of medical income with few-cluster inference**

| Effect | Inference method | Estimate, pp | 95% CI, pp | P value | Clusters/observations |
| --- | --- | --- | --- | --- | --- |
| Immediate 2019 level change | Province-clustered HC1 with t(3) | -0.18 | -1.24 to 0.88 | .623 | 4/35 |
| Immediate 2019 level change | Wild cluster bootstrap-t, null imposed, Webb weights, equal-tailed | -0.18 | -0.85 to 1.53 | .650 | 4/35 |
| Annual trend change after 2019 | Province-clustered HC1 with t(3) | 1.07 | 0.16 to 1.99 | .034 | 4/35 |
| Annual trend change after 2019 | Wild cluster bootstrap-t, null imposed, Webb weights, equal-tailed | 1.07 | -0.22 to 3.47 | .055 | 4/35 |

Note. Wild cluster bootstrap-t used null imposition, Webb weights, equal-tailed confidence intervals, and 9,999 replications. With only 4 availability-selected province clusters, all inference is exploratory.

**Supplementary Table S17. Literature-based reality check and concurrent reforms relevant to the 2019 breakpoint**

| Policy/milestone | Quantitative benchmark | Scope | Interpretive relevance | Reference |
| --- | --- | --- | --- | --- |
| Initial 4+7 pilot | 25 molecules; about 2% of hospital-channel sales in pilot cities; mean bid-price reduction 55% | Pilot-city hospital channel; conference abstract | Shows limited initial aggregate coverage; not a national spending share | 22 |
| First 3 national rounds, 2020 | 112 medicines; mean price reduction 54%; actual selected-drug volume 2.4 times committed volume | National procurement program | Coverage and volume were expanding during the study breakpoint period | 26 |
| First 6 national rounds, by 2022 | 234 medicines; involved purchasing amounts equal to about 30% of annual drug procurement by public medical institutions; mean price reduction 53% | Cumulative official program statistic | A substantial but incomplete share of all drug procurement; not a batch-specific effect size | 23 |
| Zero-markup reform | Drug expenditure per visit -6.5%; per admission -9.5% in a nationwide analysis | Earlier public-hospital financing reform | Can generate pre-2019 curvature and a lower starting drug share | 1 |
| DRG/DIP payment reform | 30 DRG pilot cities named in 2019; 71 DIP pilots by 2020 | Concurrent payment-system reforms | National timing overlaps the ITS breakpoint | 24, 26 |
| Guangzhou DIP evaluation | Total expenditure per case -3.5%, mainly through lower drug expenditure | 2018 local DIP; difference-in-differences study | Demonstrates that payment reform can change drug costs at a magnitude relevant to interpretation | 25 |
| Insurance-list negotiation, 2020 | 119 medicines newly listed after negotiation; mean negotiated price reduction 50.6% | National medical-insurance drug-list reform | Expanded access and product mix could affect all-drug costs independently of NVBP | 26 |
| Government accounting system | Effective January 1, 2019; revised accounts and hospital statements | Hospital revenue measurement | Creates a coincident classification break for revenue outcomes | 21, 28 |

Note. Public sources do not provide a validated batch-by-batch percentage of total national public-hospital drug expenditure. The 2% and 30% benchmarks have different scopes and are presented as lower-coverage and later cumulative anchors, not as directly comparable effect estimates.

# 2 Supplementary figures

**Figure S1.** Sensitivity analyses of annual trend changes in public-hospital drug-cost burden.

**Figure S2.** Exploratory regional heterogeneity in annual trend changes in public-hospital drug-cost burden.

**Figure S3.** Sensitivity analyses of the annual trend change in drug income as a share of medical income.

**Figure S4.** Observed earliest-to-latest change in drug income as a share of medical income in each exact-scope province series.

Figure S5. Sensitivity of annual trend-change estimates to pretrend functional form. Points show estimates and bars show 95% confidence intervals. Cost estimates are in CNY per year; share estimates are in percentage points per year. The dashed line marks no trend change.

Figure S6. Drug and constructed non-drug cost trajectories in 31 provinces, 2011-2024. Values are unweighted annual provincial means indexed to 2011 = 100. Non-drug cost equals total medical cost minus drug cost; the vertical dashed line marks 2019.
